# Clinical Peri-Implant Parameters and Marginal Bone Loss for Early Mandibular Implant Overdentures: A Follow-Up of 60 Months

**DOI:** 10.3390/medicina60040588

**Published:** 2024-03-31

**Authors:** Abdulaziz A. AlHelal, Abdulaziz A. Alzaid, Saad H. Almujel, Mohammed Alsaloum, Khalid K. Alanazi, Ramzi O. Althubaitiy, Khulud A. Al-Aali

**Affiliations:** 1 Department of Prosthetic Dental Sciences, College of Dentistry, King Saud University, P.O. Box 21069, Riyadh 11475, Saudi Arabia; salmujel@ksu.edu.sa; 2 Restorative and Prosthetic Dental Science Department, College of Dentistry, King Saud Bin Abdulaziz University for Health Sciences, Riyadh 11426, Saudi Arabia; zaidab@ksau-hs.edu.sa (A.A.A.); saloumm@ksau-hs.edu.sa (M.A.); 3 King Abdullah International Medical Research Center, Riyadh 11426, Saudi Arabia; 4 Conservative Dental Science Department, College of Dentistry, Prince Sattam Bin Abdulaziz University, Al-Kharj 11942, Saudi Arabia; kk.alanazi@psau.edu.sa; 5 Department of Prosthodontics, College of Dentistry, Prince Sattam Bin Abdulaziz University, Al-Kharj 11942, Saudi Arabia; r.althubaitiy@psau.edu.sa; 6 Department of Clinical Dental Sciences, College of Dentistry, Princess Nourah Bint Abdulrahman University, P.O. Box 84428, Riyadh 11671, Saudi Arabia; kaalaali@pnu.edu.sa

**Keywords:** dental implant, overdenture, implant loading, edentulous mandible, peri-implantitis

## Abstract

*Background and Objectives*: Despite the identified benefits of early implant loading, studies have questioned its advantages compared to delayed loading in edentulous patients. This study aimed to evaluate clinical peri-implant parameters and marginal bone loss around early placed and loaded mandibular implant overdentures with a 60-month follow-up. *Materials and Methods*: In this prospective cohort study, 43 patients were enrolled to receive 86 early loading sub-crestal dental implants through prosthetic guides. Implant overdentures were supported by two isolated implant locator attachments between two mental foramens. Clinical peri-implant parameters, including plaque index (PI), bleeding index (BI), peri-implant pocket depth (PIPD), and marginal bone loss (MBL) were evaluated using standardized techniques at 1, 12, 24, 36, 48, and 60 months follow-up. At 60 months, complications associated with implant overdentures (IOD’s) were noted. The mean comparison of peri-implant clinical parameters was performed through ANOVA test. A *p*-value of ≤0.05 was taken as significant. *Results*: Out of the total 43 enrolled patients, 8 patients were lost during follow-up; as a result, 35 patients completed the 5 years follow-up. The mean values of PI, BI, and PIPD increased with no statistical difference (*p* > 0.05). For marginal bone loss, an increase in the mean values was noted at different time intervals with statistical differences (*p* < 0.001). The most common complications noted were loosening of the abutment, occlusal adjustment, retentive locator loosening and replacement, and relining of the denture. *Conclusions*: Early placement of IODs failed to prevent bone loss over time and was associated with complications, predominantly consisting of abutment loosening, occlusal adjustments, broken retentive locator components, relining, and rebasing.

## 1. Introduction

Conventional complete dentures (CD) have been used as the preferred prosthetic replacement option for edentulous patients. However, utilizing CD is not without problems such as poor stability and retention, reduced chewing efficiency, speech difficulties, and bone resorption. To overcome such challenges, dental implants have gained widespread popularity as an excellent option for replacing missing teeth, primarily due to their high success rates and positive patient satisfaction levels [1]. According to the York and McGill consensus, conventional dentures are deemed inadequate for prosthodontic treatment of the edentulous mandible [2]. Substantial evidence overwhelmingly supports the notion that implant overdentures (IOD) are now considered the optimal choice for restoring the edentulous mandible [3]. Among the array of prosthetic replacements available, the IOD stands out as the most suitable option.

Implant overdentures, whether classified as implant-supported or implant-retained, offer substantial advantages over conventional complete dentures by providing enhanced stability, improved retention, and increased chewing efficiency, effectively addressing limitations associated with traditional full arch dentures [4]. However, it is crucial to acknowledge potential limitations, including the need for surgical procedures, financial considerations, and the risk of complications like peri-implantitis. Additionally, not all patients may be suitable candidates for implant therapy, considering systemic health issues or insufficient bone volume, necessitating careful case-by-case evaluation [2]. The placement of implant overdentures, whether early or delayed, plays a significant role in the overall success rate of the prosthesis [3]. Different studies have shown that delayed placement of IODs is associated with bone loss at the site of extraction, impacting the placement of implants [5]. Furthermore, alterations in the soft tissue contour have also been encountered in delayed placement, along with a psychological impact on the patient [6].

Early placement of implants is widely accepted for both removable and fixed prosthetic appliances due to higher acceptance and improved survival rates [7]. This approach is particularly important as it reduces the period of edentulism. Completely edentulous patients experience various consequences, including masticatory problems, impaired speech, compromised facial aesthetics, and psychological distress [8]. Primary implant stability is crucial for the success of implant overdentures, influencing subsequent osseointegration. Optimal stability at the time of placement minimizes micromotion and supports successful healing. In early loaded cases, where functional loads are applied soon after implant placement, achieving adequate primary stability is pivotal. Factors such as surgical technique, implant design, and bone quality significantly impact primary stability. Emphasizing the importance of precise planning and adherence to surgical protocols is essential for maximizing long-term success and patient satisfaction [3].

Despite the identified benefits of early loading, studies have questioned its advantages compared to delayed loading in edentulous patients [9]. One study reported a lower survival rate for mandibular overdentures with a single implant when early loading was performed [9]. Additionally, another study found that two-implant-supported overdentures had similar survival rates regardless of early or delayed loading of the implants [10].

Esposito et al. [11] concluded that the evidence for early loading of implant overdentures is limited and recommended to conduct well-designed studies to generate robust evidence. Moreover, numerous systematic reviews have highlighted the lack of long-term treatment outcome for early loaded implant overdentures in clinical trials as a significant limitation [12,13]. Further research is necessary to fully understand the benefits and drawbacks of early loading in implant overdenture treatment. Nonetheless, early placement of implants remains a valuable option for improving outcomes and the quality of life for completely edentulous patients. Achieving stable implant placement represents a cornerstone for the lasting success of dental implant rehabilitations. This principle holds even greater significance when navigating the complexities of limited bone volume and quality. Optimizing primary stability directly translates to superior implant outcomes in the medium- and long-term. By prioritizing this critical step, we can ensure predictable and enduring results, even in challenging scenarios with compromised bone tissue [14].

It is critical to evaluate early loaded implant overdenture treatment effectiveness, patient satisfaction, performance, stability, and long-term outcomes. The working hypothesis assumes that there will be no significant difference in the peri-implant clinical parameters and marginal bone loss (MBL) for early loaded implant overdenture over a follow-up of 60 months. This study aimed to evaluate clinical peri-implant parameters and marginal bone loss around early placed and loaded mandibular implant overdenture over a 60-month follow-up.

## 2. Materials and Methods

### 2.1. Study Design and Consent

The present clinical study was conducted in accordance with the declaration of Helsinki and its modifications (2013). The trial protocol was reviewed and approved by the Ethics Committee of Center for specialist dental practice and research center (UDCRC-082-17). Before recruitment, the participants were informed about the nature of the study and provided with all the relevant information. The patients were enrolled in this study after obtaining their verbal and written consent. The sample size was calculated using the OpenEPI sample size calculator, considering a 95% confidence interval and a test power of 80%. Based on a mean radiographic bone loss of 0.89 ± 0.74 [10], the estimated sample size was determined to be 34 patients.

### 2.2. Eligibility Criteria

Inclusion criteria comprised patients with chronic displeasure and complications with failure of existing removable partial dentures. Patients with a recent tooth extraction (12–16 weeks) and denture complaints related to the lack of retention, stability, and support were included. Patients with adequate complete dentures, free of deficiencies, were also included. Exclusion criteria comprised of patients with combined endodontic–periodontics lesions, uncontrolled Type 2 diabetes, Type V and VI bone type (Cawood and Howell classification) [15], bisphosphonates, limited mouth opening (15–20 mm), need for bone grafting, moderate to severe xerostomia, osteoporosis, previous history of cancer or reconstruction, chemotherapy, and radiotherapy.

Included patients had adequate bone height and volume for 4.1 × 10 or 4.1 × 12 mm Roxolid implant (Straumann Holding, Basel, Switzerland), evaluated with digital Orthopantomogram (OPG) (Planmeca, OY, Helsinki, Finland). Included patients kept their existing maxillary dentures and were provided with new mandibular IODs supported by two implants. The patients were treated at specialist dental practices, in Riyadh, Saudi Arabia, and the trial lasted from 2017 to 2022.

### 2.3. Surgical Procedures

All implants were placed using a surgical positioning and placement stent (copy of existing denture), by a board-certified prosthodontist, trained in implant surgery. The implants were placed using the implant surgery protocol of 16 weeks or beyond after extraction. Surgery comprised of sterile procedure with local anesthesia, mid-crestal incision, mucoperiosteal flap, osteotomy (using the protocol recommended), implant placement (inter foramina) at canine location, submerged implant placement, torqued fixture (Straumann Holding, SLActive, Basel, Switzerland) subcrestal, appropriate healing abutment, and interrupted absorbable sutures (Vicryl, Ethicon, Johnson & Johnson, New Brunswick, NJ, USA). Implants among patients included 4.1 × 12 mm (Straumann Holding, Basel, Switzerland) in 23 patients (n = 46) and 3.3 × 10 mm (Straumann Holding, Basel, Switzerland) in 12 patients (n = 24).

Existing mandibular dentures were relieved post-surgery (adjusted fitting surface) and locator abutments (Straumann Holding, Basel, Switzerland) were torqued (35 Ncm) at 6 weeks of healing. After the placement of the metal housing and picking up the housing retentive cap on the locators, existing mandibular dentures were relined to pick up the metal housing with retentive housing caps in vivo. The dentures were relined to the appropriate acrylic surface and impression retentive housings were replaced with final retentive housings in the IODs. The procedure was performed by a board-certified prosthodontist. The pickup and reline were performed in occlusion with the maxillary denture. Early implant loading refers to the placement of prosthetic components, such as dentures, on dental implants shortly after their surgical placement in the jawbone.

### 2.4. Measuring Clinical Peri-Implant Parameters

**Figure 1:**
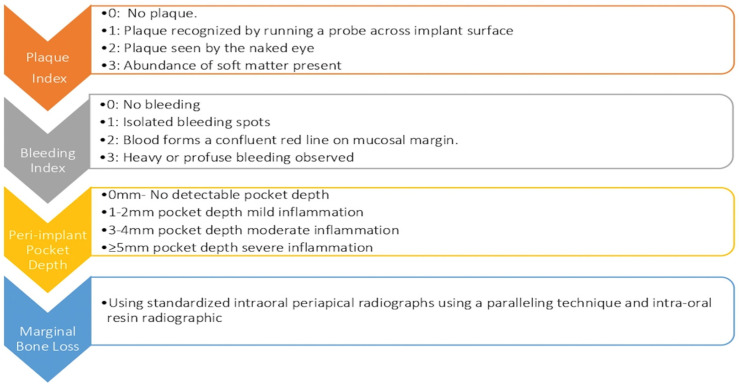
Schematic diagram of indexes used to measure peri-implant clinical parameters.

All implants and mandibular IODs were evaluated for PI, BI, PIPD, and MBL at 1, 12, 24, 36, 48, and 60 months. The unit of analysis in this study was per implant, the outcome was reported for each individual implant site. Each dental implant was treated as a separate unit in the statistical analysis. Plaque index [16], bleeding index [17], and peri-implant pocket depth [18] reference scorings are presented in Figure 1. Marginal bone levels around the implants were monitored with standardized intraoral periapical radiographs using a paralleling technique and intra-oral resin radiographic digital sensor placement jig for each patient. The radiographic bone levels were evaluated on the Planmeca Romexis Dental Software 4.0 (Planmeca©, Helsinki, Finland). Images were calibrated and bone levels were assessed from the implant abutment junction to the crest of bone mesial and distal to the implant. Evaluations were performed at 1, 12, 24, 36, 48, and 60 months. For all evaluations, a mean and standard deviation was identified. The prosthodontist evaluated complications and failures related to denture, abutment, and implants. The frequency of these complications was reported after a 60-month follow-up period. The assessment included various parameters such as abutment loosening, fracture, wear, implant fracture, occlusal adjustment, retentive cap looseness, retentive cap loss, replacement, metal housing looseness, and loss. Additionally, the occurrence of denture fractures, relines, rebases, and replacements was also observed.

### 2.5. Statistical Analysis

Data were analyzed using a statistical program for social sciences (SPSS, Version 25, IBM, Armonk, NY, USA). Normality distribution was identified with the Kolmogorov–Smirnov test. Two-way ANOVA was applied for the comparison of PI, BI, PIPD, and MBL. The Tukey’s honestly significant difference (HSD) test was conducted to assess pairwise differences in marginal bone loss (MBL) at various time intervals. A *p*-value of <0.05 was considered statistically significant.

## 3. Results

**Figure 2:**
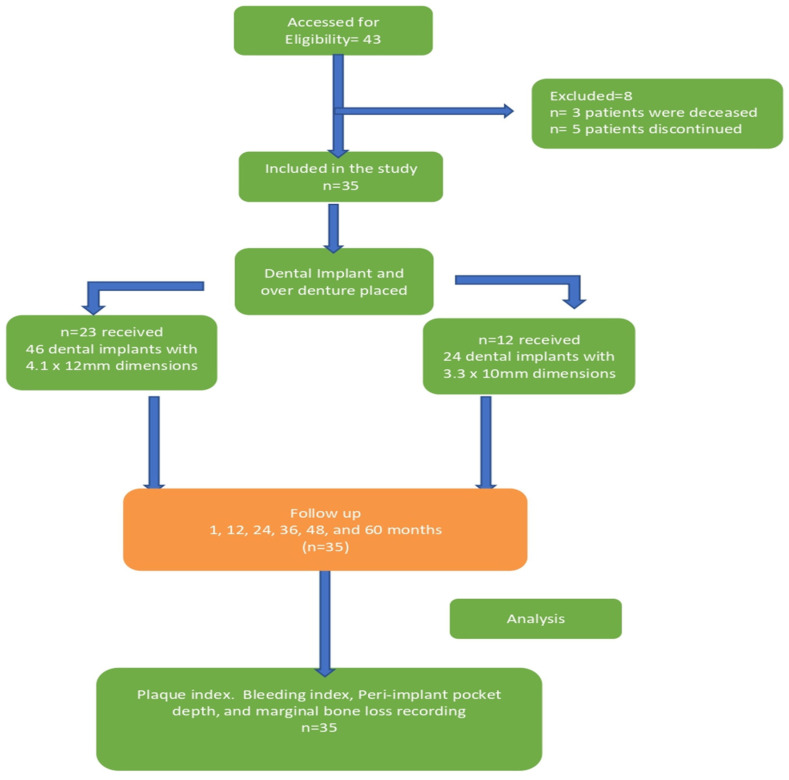
Flow diagram of study methodology.

At the beginning of the study, forty-three patients were initially included. However, throughout the course of the study, three patients passed away, and five patients discontinued their participation. As a result, the final assessment and evaluation included a total of 35 patients, as depicted in Figure 2, encompassing all the relevant evaluations and parameters.

Table 1 presents the general characteristics of the included study patients. The mean age of the participants was 63.7 ± 6.1. The gender distribution was as follows: 23 males and 12 females. The mean age of denture wearing was 3.8 ± 1.6. The peri-implant parameters, including plaque index, bleeding index, and peri-implant pocket depth, were assessed at various time intervals: 1 month, 12 months, 24 months, 36 months, 48 months, and 60 months. A comparison was made between these characteristics and the baseline values (controls).

In relation to the PI, a notable decrease in the mean value was observed from baseline (1.1 1 ± 0.5) to 36 months (0.89 ± 0.6). For the 36 months and 60 months evaluations, PI increased from 0.89 ± 0.6 to 1.54 ± 0.6, respectively. Interestingly, PI did not show significant change throughout the follow-up duration from 1 month to 60 months (*p* > 0.05). There was an increase in the BI from baseline (0.84 ± 0.6) to 60 months (1.07 ± 0.7) follow-up, but the difference was not statistically significant (*p* > 0.05). There was a gradual increase in PIPD from baseline (3.08 ± 1.3) to 60 months (4.34 ± 1.0) follow-up, the difference was statistically significant (*p* < 0.05), as shown in Table 2.

The marginal bone loss (MBL) at each time interval was compared with the baseline (control). MBL at baseline was (−0.5 ± 0.3), which significantly increased at 48 months (1.7 ± 0.6) and 60 months (2.3 ± 1.0) follow-up. Overall, MBL significantly increased (*p* < 0.01) from baseline to 60 months follow-up (Table 3).

The Table 4 summarizes the findings from the Tukey’s Honestly Significant Difference (HSD) test. Statistically significant differences were observed between 1 month and 12 months, 36 months and 48 months, 48 months and 60 months, as well as 1 month and 60 months. These results suggest distinct patterns of MBL changes over time, where significant alterations in marginal bone levels occurred during the 5-year study period.

Various prosthetic complications were observed over a 5-year period. Concerning abutment-related complications, 21 implants experienced abutment loosening, while wear was observed in 14 abutments and occlusal adjustments were performed in 23 cases. There were no reported cases of abutment or implant fractures. Additionally, complications related to retention elements were noted during follow-up visits. In 13 cases, the metal housing used for retention became loose. The retentive locator component was reported as broken in 20 cases and loose in 33 cases. Replacement of the retentive locator component was required in 33 cases. Furthermore, complications associated with the IODs were also observed. Sixteen cases required a reline, and four cases required rebasing. Fractures of the IODs were observed in five cases, and new dentures were used to replace five IODs, as presented in Table 5.

## 4. Discussion

Implant-supported overdentures have gained popularity as an effective treatment option, particularly in cases where conventional complete dentures have shown limitations [19,20]. The present study aimed to investigate peri-implant parameters and prosthetic complications of IODs over a 5-year follow-up. It was hypothesized that no significant difference in the peri-implant clinical parameters and MBL will be present for early loaded IOD over a 5-year follow up. The hypothesis was partly rejected as MBL significantly increased from baseline to 60 months; however, PI, BI, and PIPD showed comparable outcomes.

Implant-supported prostheses have been widely embraced by patients, particularly in cases where traditional complete dentures have proven inadequate [19]. Implant overdentures provide various advantages over conventional dentures such as improved stability and retention, significant improvement in chewing efficiency, preservation of bone health by overcoming bone loss, and greater aesthetic outcomes. Moreover, patients tend to maintain their oral hygiene at acceptable levels as compared to patients with conventional dentures [13].

In the present study, PI, BI, PIPD, and MBL were assessed around early loaded IODs. The mean values of PI, BI, and PIPD increased from baseline to 5 years; however, the difference was not significant. Such results correlate with a study by Maryod et al., where they reported higher values of BI, PI, and PIPD in patients with early loading implants as compared to delayed loading [21]. However, one of the limitations of this study was the shorter duration of follow-up of the patients. Similarly, another systematic review evaluating the long-term clinical outcomes of different loading protocols of IODs reported, that although PI and PIPD values of immediate loading protocols were greater than conventional loading protocols, early loading protocols are well established and are worthy of consideration in clinical practices [22]. Favorable PI, BI, and PIPD reflects good oral hygiene, healthy peri-implant tissues, and decreased risk of complications associated with early loaded implant overdentures. This indicates that the osseointegration process has occurred successfully, ensuring a solid foundation for the prosthetic restoration. Biologically, early loading allows for functional stimulation of the implant, which promotes better bone remodeling and preserves bone density. It also helps to distribute occlusal forces, reducing the risk of implant-related complications in the long term [23].

Early loading of implant overdentures is clinically preferred as it enables patients to regain their functional and aesthetic aspects earlier as compared to delayed loading. Moreover, biologically speaking, early loading mitigates the risk of bone loss [23]. One study has reported that early loading of implants reduces the micromotions of implants [24]. However, according to Zhu et al., early loading of implant overdentures has been associated with a higher risk of implant failure due to its micromotions [25]. The variability of these results is primarily due to variability of prosthodontic and peri-implant outcomes measured in different studies.

One of the vital indicators of success rates of overdentures are the levels of marginal bone around implants [22]. Based on the findings of the current study, there was a significant increase in the mean MBL from baseline to 60-month follow-up period (from −0.5 ± 0.3 to 2.3 ± 1.0). Despite this observed bone loss over time, it remained within an acceptable range. It is worth noting that early bone loss of 1.5 mm within the first two years of implant placement is generally considered acceptable [26]. On the contrary, a systematic review conducted by Sanda et al., comparing immediate and conventional loading implants, concluded that no significant difference in bone loss was reported [27]. By contrast, Pardal et al., characterized MBL as a risk factor for early implant loss when comparing early and delayed placement protocols [28].

In the present study, we chose subcrestal surgical technique and implant placement and implants were submerged below the level of crestal bone [29]. A study by Pellicer-Chover concluded that crestal and subcrestal dental implant placement techniques are used in IODs; however, it did not show any effect [30]. Moreover, de Siqueira and colleagues evaluated the impact of different types of dental implants over a period of 5 years on crestal bone levels [31]. They found no difference in marginal bone levels between subcrestal and crestal implant placement; however, subcrestal positioning resulted in higher levels of bone. A systematic review and meta-analysis concluded that subcrestal implant placement resulted in fewer changes in terms of MBL as compared to crestal placement [32]. A study by Degidi et al. concluded that subcrestal implant placement provided favorable outcomes in bone preservation as compared to crestal implant placement [33]. Other than the placement of dental implants, mucosal thickness also plays a vital role in the impact of bone loss around the dental implant [34].

The implant overdentures when placed in the oral cavity are subjected to an array of insults of which masticatory forces are the most significant cause of insult. Due to such masticatory forces, various post-operative complications can be encountered in IODs in terms of abutments, retentive components, and dentures [22,27]. In our study, we found loosening of the abutment, occlusal adjustment of the abutment, retentive locator loosening, replacement of retentive locator, and relining of the dentures as common complications. Similar and other complications have been reported by Ulku and colleagues, where abutment loosening, loss of retention, mucositis, and fracture of abutment were reported [35]. Other than the complications, patients with IODs reported suffering from immediate post-operative symptoms such as pain, swelling, bleeding, pain on chewing, and discomfort [36].

Implant-supported overdentures are a beneficial treatment option for completely edentulous patients, especially in situations where conventional complete dentures have failed [35,37]. Therefore, according to the results of our study, IODs must be given consideration in clinical practice. In order to retain and stabilize IOD’s, precision attachments are recommended over the implant abutments [34]. A wide range of attachment systems are employed in implant overdentures (IODs). The present study adopted the isolated locator type of attachment system. The ball-based attachment systems are also recommended due to their cost-effectiveness and ease of maintenance; however, for better function, the implant angulation should be given consideration [37]. Additionally, bar-based attachment systems provide optimal retention and implant splinting, but the cost and prosthetic technique sensitivity are their major drawbacks encountered in clinical practice [38]. Magnetic attachment systems are rarely used as corrosion and loss of magnetism significantly reduces its long-term utilization in IODs [39].

This study had some inherent shortcomings such as the use of one type of attachment system. The evaluation of different types of attachment systems can increase the scope of future similar studies. Secondly, the accuracy and reliability of measuring PI, BI, MBL, and PIPD may vary between different observers. Moreover, a follow-up period longer than 60 months can provide a comprehensive understanding of the stability and potential long-term challenges with IODs. The present study employed the use of bleeding index for dental implants instead of bleeding on probing (BOP). The bleeding index may not accurately reflect the specific condition of the peri-implant tissues. The bleeding index is a more general measure of gingival health and may not be sensitive enough to detect localized inflammation or pathology around dental implants. BOP, on the other hand, is a more specific and direct measure of inflammation at the implant site, providing a more accurate assessment of peri-implant health. A notable restraint of the study is the absence of explicit consideration and evaluation of the influence of keratinized gingival tissue on marginal bone loss (MBL). Given the recent findings suggesting a relationship between the presence of keratinized tissue, MBL, and depth of fixture insertion [32], the study missed an opportunity to explore this important parameter. The role of keratinized gingiva in peri-implant health is significant, impacting inflammation levels and potentially affecting MBL outcomes. The lack of data on this specific aspect limits the comprehensive understanding of factors contributing to MBL in the studied population. Therefore, future research could benefit from incorporating detailed assessments of keratinized gingival tissue and its potential correlations with MBL, providing a more comprehensive perspective on peri-implant tissue health [40].

Despite the limitations of the study, the study had notable strengths. The longitudinal design was employed, enabling the assessment of clinical peri-implant parameters spanning a 5-year timeframe. This approach revealed satisfactory values for PI, BI, MBL, and PIPD. However, it is important to note that IODs with early implants exhibited moderate prosthetic complications, emphasizing the necessity for diligent maintenance in the treatment phase for patients utilizing IODs. In the future, conducting studies with larger sample sizes, involving multiple centers, extending follow-up periods, and performing comparative analyses would significantly contribute to enhancing the evidence base, improving patient care, and optimizing treatment outcomes in the field of implant prosthesis.

## 5. Conclusions

Early placement of IODs failed to prevent bone loss over time and were associated with complications, predominantly consisting of abutment loosening, occlusal adjustments, broken retentive locator components, relining, and rebasing.

## Figures and Tables

**Table 1:** General characteristics of patients included (n = 35).

Characteristics	Mean	SD
Age	63.7	6.1
Gender (M/F)	23/12	
Age of denture wearing	3.8	1.6

SD, standard deviation.

**Table 2:** Peri-implant soft tissue parameters from baseline (control) to 5 years function (two-way ANOVA test).

Soft Tissue Parameters	Baseline (1 Month) (Mean + SD)	12 Months (Mean + SD)	24 Months (Mean + SD)	36 Months (Mean + SD)	48 Months (Mean + SD)	60 Months (Mean + SD)	*p*-Value
PI	1.1 1 ± 0.5	1.3 ± 0.6	0.94 ± 0.4	0.89 ± 0.6	1.36 ± 0.5	1.54 ± 0.6	>0.05 (0.065)
BI	0.84 ± 0.6	0.65 ± 0.6	0.68 ± 0.5	0.72 ± 0.4	1.05 ± 0.5	1.07 ± 0.7	>0.05 (0.366)
PIPD	3.08 ± 1.3	2.50 ± 0.9	2.78 ± 0.7	3.40 ± 1.1	3.36 ± 1.0	4.34 ± 1.0	<0.05 (0.004)

PI: plaque index, BI: bleeding index, PIPD: peri-implant pocket depth.

**Table 3:** Peri-implant bone levels from baseline (control) to 5 years function using ANOVA test.

Clinical Parameters	Baseline (1 Month) (Mean + SD)	12 Months (Mean + SD)	24 Months (Mean + SD)	36 Months (Mean + SD)	48 Months (Mean + SD)	60 Months (Mean + SD)	*p*-Value
MBL	−0.5 ± 0.3	0.50 ± 0.3	1.3 ± 0.4	1.40 ± 0.5	1.7 ± 0.6 ^a^	2.3 ± 1.0 ^a^	* <0.001

MBL: marginal bone loss, * ANOVA, superscripted alphabet ^a^ denotes significant difference from baseline.

**Table 4:** Comparison of marginal bone loss over time, post-hoc analysis (Tukey’s test).

Comparison	Mean Difference (MBL)	*p*-Value
1 month vs. 12 months	0.50	0.003 *
12 months vs. 24 months	0.10	0.312
24 months vs. 36 months	0.10	0.998
36 months vs. 48 months	0.30	0.033 *
48 months vs. 60 months	0.60	0.016 *
1 month vs. 60 months	2.80	0.001 *

* Statistical significance MBL: marginal bone loss.

**Table 5:** Prosthetic complication frequency among IODs over 5 years.

Types of Complications	Frequency and Percentage (n = 86 Implants)
Abutment (A)	
A. Loosening	21 (24.42%)
A. Wear	14 (16.28%)
A. Fracture	-
Implant fracture	
Occlusal adjustment	23 (26.74%)
Retention Element	
Metal housing loose	13 (15.12%)
Retentive locator broken	20 (23.26%)
Retentive locator loose	33 (38.37%)
Replacement of retentive locator	33 (38.37%)
Denture (n = 35 IOD)	
Reline	16 (18.60%)
Rebase	4 (4.65%)
Fracture	5 (5.81%)
IOD replacement	5 (5.81%)

## Data Availability

The data are available on contact from the corresponding author.
